# Cross Pharmacological, Biochemical and Computational Studies of a Human Kv3.1b Inhibitor from *Androctonus australis* Venom

**DOI:** 10.3390/ijms222212290

**Published:** 2021-11-13

**Authors:** Sonia Maatoug, Amani Cheikh, Oussema Khamessi, Hager Tabka, Zied Landoulsi, Jean-Marie Guigonis, Sylvie Diochot, Saïd Bendahhou, Rym Benkhalifa

**Affiliations:** 1 Laboratoire Biomolécules, Venins et Applications Théranostiques (LR20IPT01), Institut Pasteur de Tunis, Université Tunis El Manar, 13 Place Pasteur BP74, Tunis 1002, Tunisia; cheikhamani@gmail.com (A.C.); tabkahager@yahoo.fr (H.T.); zied.landoulsi@uni.lu (Z.L.); 2 Laboratoire des Biomolécules Thérapeutiques, Institut Pasteur de Tunis, Université de Tunis El Manar, 13 Place Pasteur BP74, Tunis 1002, Tunisia; oussama.khamassi@pasteur.tn; 3 Faculté des Sciences de Bizerte, Université de Carthage, Bizerte 7021, Tunisia; 4 Laboratory Transporter in Imaging and Radiotherapy in Oncology (TIRO), Direction de la Recherche Fondamentale (DRF), Institut des Sciences du Vivant Fréderic Joliot, Commissariat à l′Energie Atomique et aux Énergies Alternatives (CEA), Université Côte d’Azur, F-06107 Nice, France; Jean-Marie.GUIGONIS@univ-cotedazur.fr; 5 Institut de Pharmacologie Moléculaire et Cellulaire, Centre National de la Recherche Scientifique, Université Côte d’Azur, 660 Route des Lucioles, Sophia-Antipolis, 06560 Valbonne, France; diochot@ipmc.cnrs.fr; 6 UMR7370 CNRS, LP2M, Université Côte d’Azur, Labex ICST, Nice, France; said.bendahhou@univ-cotedazur.fr

**Keywords:** Kv3.1 channel, *Androctonus australis hector* venom, open channel blocker, alpha-KTx

## Abstract

The voltage-gated K^+^ channels Kv3.1 display fast activation and deactivation kinetics and are known to have a crucial contribution to the fast-spiking phenotype of certain neurons. AahG50, as a natural product extracted from *Androctonus australis hector* venom, inhibits selectively Kv3.1 channels. In the present study, we focused on the biochemical and pharmacological characterization of the component in AahG50 scorpion venom that potently and selectively blocks the Kv3.1 channels. We used a combined optimization through advanced biochemical purification and patch-clamp screening steps to characterize the peptide in AahG50 active on Kv3.1 channels. We described the inhibitory effect of a toxin on Kv3.1 unitary current in black lipid bilayers. In silico, docking experiments are used to study the molecular details of the binding. We identified the first scorpion venom peptide inhibiting Kv3.1 current at 170 nM. This toxin is the alpha-KTx 15.1, which occludes the Kv3.1 channel pore by means of the lysine 27 lateral chain. This study highlights, for the first time, the modulation of the Kv3.1 by alpha-KTx 15.1, which could be an interesting starting compound for developing therapeutic biomolecules against Kv3.1-associated diseases.

## 1. Introduction

Ion channels are transmembrane proteins that regulate the flow of ions across biological membranes and Kv3.1 channels are one of the Shaw-type voltage-gated K^+^ channels that are abundantly expressed in fast-firing neurons such as cortical interneurons, hippocampal interneurons, corticothalamic neurons, auditory brain stem neurons and GABAergic inhibitory neurons [1,2,3,4,5,6].

Kv3.1 has an important role in the synchronization of cortical circuits and the generation of brain rhythms, and it is crucial for perception, alertness, learning, control of the sleep cycle and motor activity [7,8].

It is evident that Kv3.1 is functionally relevant in adult Neural Progenitor Cell (NPC) expansion and neuronal lineage commitment [9,10] and we have also confirmed its role during 1C11 cell line differentiation, which is used as an in vitro model for serotonergic release [11]. The pharmacological and genetic disruption of Kv3 currents leads to impaired fast spiking in inhibitory neurons and increased seizure susceptibility which explains why Kv3.1 channel loss of function causes many physiological disorders such as ataxia, myoclonus, tremor, hyperactivity and reduction in sleep time [7,8], in addition to alterations in synaptic transmission at the parallel fibre–Purkinje cell synapses [12].

Unfortunately, few pharmacological tools have been reported as modulating Kv3.1 current. Modulators such as tetraethylammonium (TEA), Fluoxetine or gambierol are nonspecific and are functionally limited [13,14]. More recently, Brown and collaborators reported about two imidazolidinedione derivatives AUT1 and AUT2, that modulate Kv3.1 current by increasing the open probability of the channels [15]. Besides, venoms are a rich source of neurotoxins [16] that are able to block or modify the function of their targeted ion channels in excitable cells. Scorpion neurotoxins are classified as either long- or short-chain toxins. Short-chain toxins usually contain 30–40 residues and three or four disulfide bridges. Most of the short-chain scorpion toxins described to date block voltage-dependent K^+^ channels. Scorpion venom derived AahG50, a fraction previously isolated from the North African *Androctonus australis hector* venom, is mainly composed of Na^+^ channel toxins even if some K^+^ channels toxins were reported [17,18,19,20,21,22,23]. Indeed, in 2005, Srairi-Abid and collaborators demonstrated the presence of two toxins in AahG50, KAaH1 and KAaH2, which are active on Kv1.1 and Kv1.3 subtypes. When tested at 100 nM on Kv3.1 current, both toxins failed to affect channel activity, comforting us that different peptides in the AahG50 fraction would be responsible for the current inhibition [24]. A primary screening of *Androctonus australis hector* venom showed that, the toxic fraction, AahG50 inhibits IKv3.1 in a dose and potential-dependent manner with IC50 = 40.4 µg/mL, without modifying the activation threshold [24].

The present work describes a novel Kv3.1 inhibitor peptide isolated from *Androctonus australis hector* venom. The research study is based on advanced biochemical analysis combined with pharmacological characterization using a multitude of techniques in electrophysiology besides High Resolution Mass Spectrometry and computational, in silico studies based on molecular docking. We identified alpha-KTx 15.1 as the active peptide. It occludes Kv3.1 channel pore by the means of the lysine (Lys27) lateral chain. The alpha-KTx 15.1 belongs to the alpha-KTx family and presents 93% of homology with AmmTX3, which is a specific Kv4 channel blocker. Our results provide new insight into the possible targets of alpha-KTx 15.1 on Kv3.1 channels, besides Kv4 channels type, which should enhance the development of new therapeutic pathways for the treatment of a variety of dysfunctions.

## 2. Results

### 2.1. AahG50 Toxic Venom Fraction of Androctonus australis hector Scorpion Blocks Human Kv3.1 Channel

**Figure 1:**
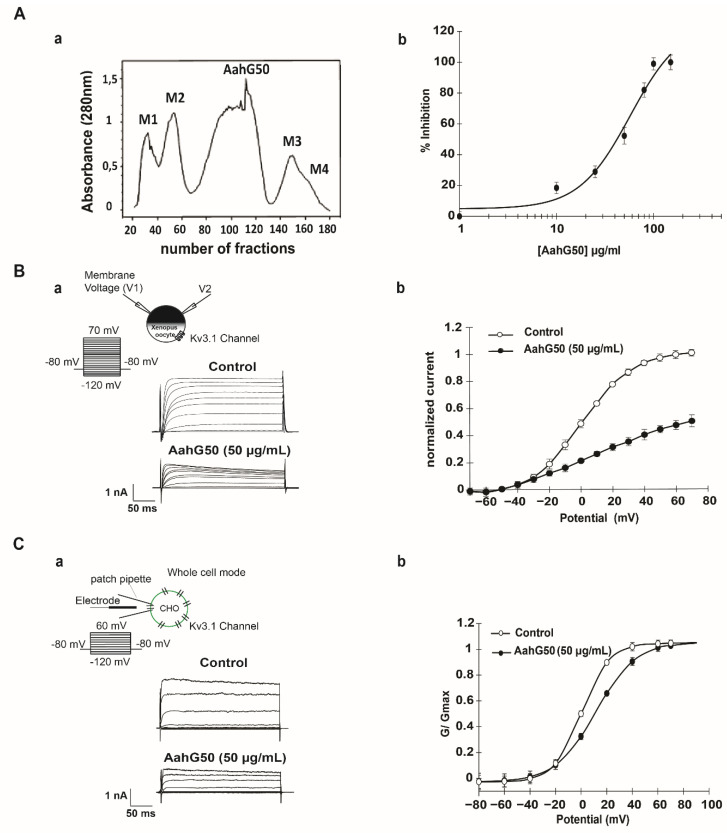
Inhibitory effect of AahG50 on Kv3.1 currents. (**Aa**)) Purification of *Androctonus australis hector* scorpion venom. Dialyzed venom loaded into a Sephadex G50 column allowed the collect 5 fractions (M1, M2, AahG50, M3 and M4). (**Ab**)) Dose–response curve of Kv3.1 channel inhibition at various concentrations of AahG50. Current amplitudes measured at the end of the depolarizing pulse (+60 mV), the percentage of inhibition plotted against respective concentrations of AahG50 (10, 25, 50, 100 and 150 µg/mL). (**Ba**)) Whole-cell currents, recorded in *Xenopus* oocytes, elicited by the application of 250 ms depolarizing pulses from −120 mV to +70 mV in 10 mV increments, from a holding potential of −80 mV, under control conditions and after the addition of 50 µg/mL AahG50. (**Bb**)) The normalized current voltage relationship of Kv3.1 currents in *Xenopus* oocytes plotted under control conditions and after the perfusion of 50 µg/mL of AahG50. (**Ca**)) Whole-cell currents, recorded in CHO cells, elicited by the application of 250 ms depolarizing pulses from −120 mV to +60 mV in 20 mV increments, from a holding potential of −80 mV, under control conditions and after the addition of 50 µg/mL AahG50. (**Cb**)) The normalized conductance-voltage relationship of Kv3.1 current in CHO cells plotted under control conditions and after the perfusion of 50 µg/mL of AahG50.

Crude Aah venom was separated by gel filtration. The resulting profile (Figure 1(Aa)) shows five partially resolved fractions; (M1, M2, AahG50, M3 and M4).

We focused our investigations on the AahG50 fraction since we have previously shown that it inhibits the Kv3.1 current [24]. Two-Electrodes Voltage Clamp (TEVC) recording illustrates that AahG50 (50 µg/mL) reduces Kv.3.1 currents (Figure 1(Ba)). The normalized current voltage (IV) curves obtained before and after addition of AahG50 at 50 µg/mL shows that the inhibition of IKv3.1 amplitude is voltage-dependent with a maximum of inhibition of 50 ± 1.3% (n = 10) at +70 mV when currents are fully activated (Figure 1(Bb)). The inhibition is sustained in presence of AahG50, reaches a steady state within 3 to 4 min, and could be reversed on washout.

Patch clamp tests, performed in the mammalian CHO cells, are not only useful in confirming the effect of AahG50 on Kv3.1 current, but also allow the use of small amounts of the tested fractions. Moreover, whole cell patch clamp tests demonstrate that AahG50 (50 µg/mL) reduces Kv3.1 current (Figure 1(Ca)). The concentration dependence of AahG50 on Kv3.1 is measured after perfusion of AahG50 from 10 to 150 μg/mL. It induces a slight inhibition about 18.4 ± 3.75% at 10 µg/mL, a maximum of inhibition is reached at 100 and 150 µg/mL with an IC50 value of 52 ± 0.3 µg/mL and Hill coefficient 1.6 ± 0.03 (n = 30), (Figure 1(Ab)).

Application of AahG50 (at 50 µg/mL) on Kv3.1 channel induced a significant rightward shift of 11.04 ± 0.13 mV in V_1/2_ on the steady state activation with a significant increase in the slope factor value (n = 30, *p* < 0.05) (Figure 1(Cb), Table 1). The threshold of activation did not yield any change and was approximately −20 mV, in control condition and after perfusion of AahG50 (50 µg/mL) (Figure 1(Cb), Table 1).

### 2.2. Pharmacological Characterization of the Component Inhibiting Kv3.1 Channel

#### 2.2.1. Whole Cell Tests with AahG50 FPLC Fractions

**Figure 2:**
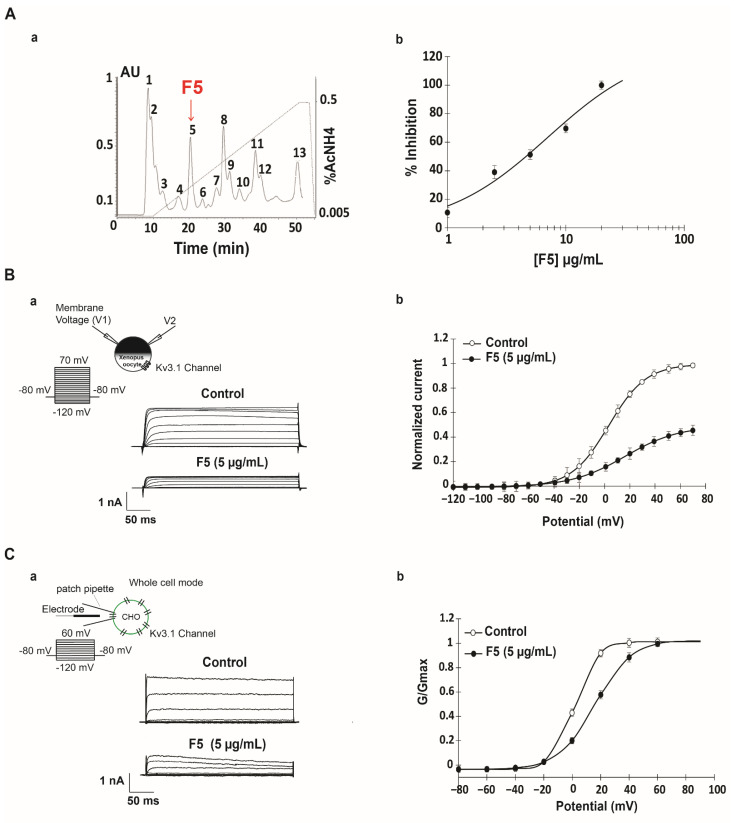
Identification of FPLC subfractions in AahG50 active on Kv3.1 channel. (**Aa**) Purification of AahG50 with FPLC. Seven milligrams of proteins were loaded per run on a cation exchange Resource S column pre-equilibrated with 0.05 M ammonium acetate buffer (pH 6.6). Proteins were eluted with a 40 min linear gradient from 0.05 to 0.5 M ammonium acetate (pH 6.6) at a flow rate of 1 mL/min; absorbance was monitored at 280 nm. (**Ab**) Dose–response curve for the inhibition of Kv3.1 by various concentrations of F5. Current amplitudes were measured at the end of the depolarizing pulse (+70 mV), the percentage of inhibition was plotted against respective concentrations of F5. The curve was fitted by the Hill equation. (**Ba**) Whole-cell currents, recorded in *Xenopus* oocytes, elicited by the application of 250 ms depolarizing pulses from −120 mV, to +70 mV in 10 mV increments, from a holding potential of −80 mV, under control conditions and after the addition of 5 µg/mL of F5 fraction. (**Bb**) The normalized current voltage relationship of Kv3.1 currents in *Xenopus* oocytes were plotted under control conditions and after the perfusion of 5 µg/mL of F5. (**Ca**) Whole-cell currents, recorded in CHO cells, using the same protocol described previously, under control conditions and after the addition of 5 µg/mL of F5. (**Cb**) The normalized conductance-voltage relationship of Kv3.1 current in CHO cells is plotted under control conditions and after the perfusion of 5 µg/mL of F5.

AahG50 fraction separated by Fast Protein Liquid Chromatography (FPLC) yields 13 subfractions from F1 to F13, as shown on the profile (Figure 2(Aa)). Each of the purified peaks was then separately tested on IKv3.1 with protocols described in Figure 2(Ba,Ca). Among AahG50 fractions only F5, eluted at 20 min, reduces Kv3.1 current, contrary to the other fractions (n = 10) (Figure 2(Ba,Ca)). The I-V curve shows that F5 at 5 µg/mL blocks Kv3.1 current in a voltage-dependent manner with a maximum effect of 50 ± 0.3% when channels are fully open (Figure 2(Bb)). The inhibition is sustained in presence of F5, reaches a steady state within 3 to 4 min, and could be reversed on washout. The concentration dependence was studied by the application of a test pulse at +60 mV in the presence of increasing concentrations of F5 from 1 to 20 µg/mL (Figure 2(Ab)). It acts in a dose dependent manner by reducing Kv3.1 current with a maximum inhibition of 100 ± 2.8% at a concentration of 20 µg/mL (Figure 2(Ab), Table 1) while IC50 and Hill coefficient are, respectively 5.45 ± 0.5 µg/mL and 1.19 ± 0.5. The activation curve shows a rightward shift of 16.42 ± 0.32 mV in V_1/2_ and an increase in the slope factor value (Figure 2(Cb) and Table 1) but the activation threshold does not change (n = 30) (Figure 2(Bb,Cb) and Table 1).

#### 2.2.2. Whole-Cell Tests with HPLC Fractions on IKv3.1

We pursued a combined optimization through “High-performance liquid chromatography” (HPLC) purification and patch-clamp screening steps to characterize peptides from F5 fraction which are candidates to block in a potent and selective manner the Kv3.1 current.

**Figure 3:**
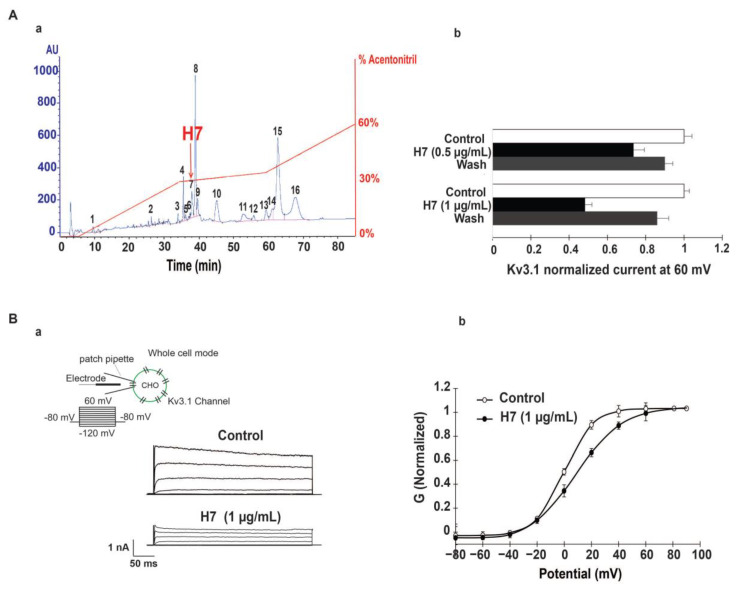
Characterization of H7 inhibitory effect on Kv3.1. (**Aa**) HPLC purification of AahG50 venom fraction (F5) obtained with FPLC method. Fifty micrograms of F5 were loaded per run on a C18 column reversed phase HPLC and 18 fractions (H1 to H18) eluted at a flow rate of 1 mL/min, using a multi-step gradient (90 min) from 0 to 60% of buffer B (0.1% TFA in CH3CN) in buffer A (0.1% TFA in water). (**Ab**) Kv3.1 normalized current at +60 mV shown before, and after perfusion of H7 at 0.5 and 1 µg/mL and after washing. (**Ba**) Whole-cell currents, recorded in mammalian cells, elicited by the application of 250 ms depolarizing pulses from −120 mV, to +60 mV in 20 mV increments, from a holding potential of −80 mV, under control conditions and after the addition of 1µg/mL of H7. (**Bb**) The normalized conductance (G) voltage relationship of Kv3.1 current plotted under control conditions and after the perfusion of 1 µg/mL of H7.

The chromatogram in Figure 3(Aa) represents the reversed phase HPLC purification of F5 fraction. The sample was fractionated on a C18 reversed phase HPLC column and gradient conditions adjusted in order to perform the full separation of F5 and to provide a good level of separation (Figure 3(Aa)). Eighteen peaks were manually collected regarding the wavelength (OD) at 214 nm at different time of retention as illustrated in the profile. Peak amplitudes are proportional to protein concentrations in the sample (Figure 3A).

The 18 fractions obtained with HPLC were examined for their ability to inhibit Kv3.1 current. Only, subfraction number 7, eluted at 37.9 min Figure 3(Aa), reduces Kv3.1 current as described in Figure 3(Ba). All other subfractions affect neither the amplitude nor the kinetics of the currents.

Using IV protocol (Figure 3(Ba)), we found that H7 reduces IKv3.1 in a concentration-dependent manner with 51 ± 0.2% and 25 ± 0.5% of inhibition at 1 µg/mL and 0.5 µg/mL, respectively (Figure 3(Bb)). The inhibition is sustained in presence of H7 and was partially reversed upon washing (Figure 3(Ab)).

H7 at 1 µg/mL diminishes Kv3.1 current amplitudes with a rightward shift and slowing down of the steady state activation curve. Indeed, H7, 1 µg/mL produces a positive shift, of 11.94 ± 0.3 mV in V_1/2_, and increases the slope factor value (Figure 3(Ab) and Table 1) but it does not modify the activation threshold (n = 20) (Figure 3(Bb) and Table 1).

### 2.3. Validation of H7 Inhibitory Effect on Kv3.1 Single Current Recording

**Figure 4:**
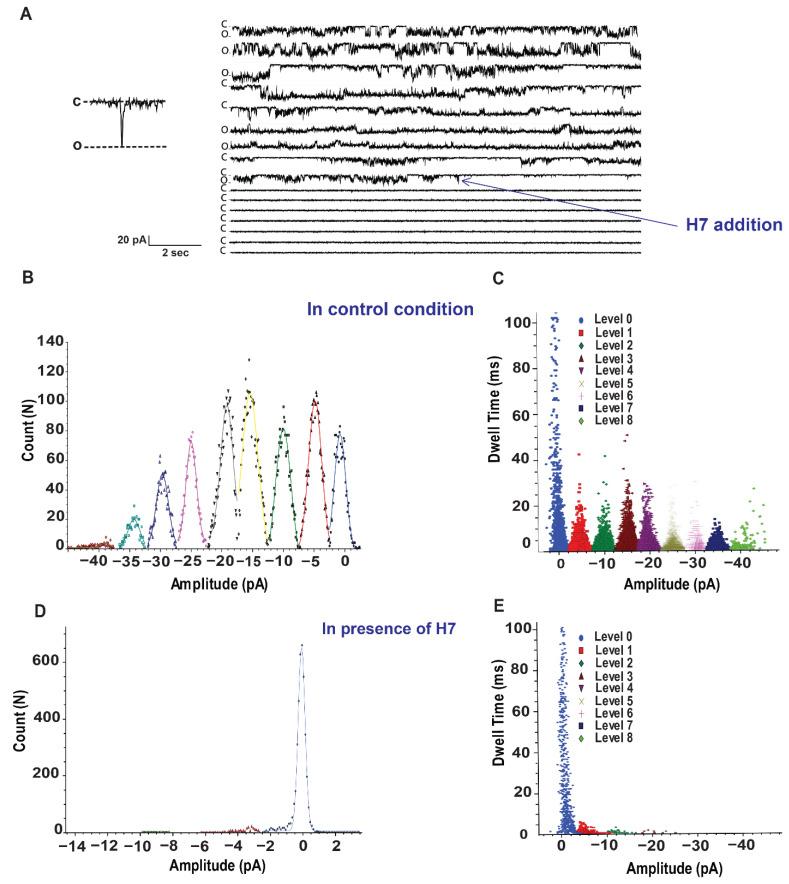
Validation of H7 inhibitory effect on Kv3.1 single current. (**A**) A representative trace of Kv3.1 channels in planar lipid bilayer from 4 min of continuous recording at −60 mV. Closed (c) and open (o) levels are indicated. H7 (0.6 µg/mL) is added at the indicated time point. (**B**) Fits of Gaussian distributions of multiple histograms led to a single channel current level of 4.74 ± 0.063 pA at −60 mV. (**C**) Dwell time of the whole 4 min recording at −60 mV revealed channel conductance levels (levels 1 to 8). (**D**,**E**) represent, respectively multiple histograms and dwell time after addition of H7.

Figure 4A shows a trace of Kv3.1 current in single channel recording. In control conditions, Kv3.1 channel openings are detected from a test potential of −60 mV. Amplitude histograms for the recording show simultaneous openings of multiple channels (Figure 4A,B). Fits of Gaussian distributions of multiple histograms led to a single channel current level of 4.74 ± 0.063 pA at −60 mV in (Figure 4B). Analysis of all events of the full 4 min recording at −60 mV revealed the presence of multiple conductance states of Kv3.1 activity (Levels 1 to 8) in (Figure 4C).

After the addition of H7 (0.6 µg/mL) at the indicated time point (A), the channel activity was abolished Figure 4D.

Kinetic analysis revealed differences in the open dwell-time distribution before (Figure 4C) and in presence of H7 at 0.6 µg/mL (Figure 4E). The most noticeable effect observed in open dwell-time distribution, is an increase in the closed channel lifetimes, but open channel levels decrease significantly and almost abolished.

### 2.4. Mass Spectrometric Analysis of H7

**Figure 5:**
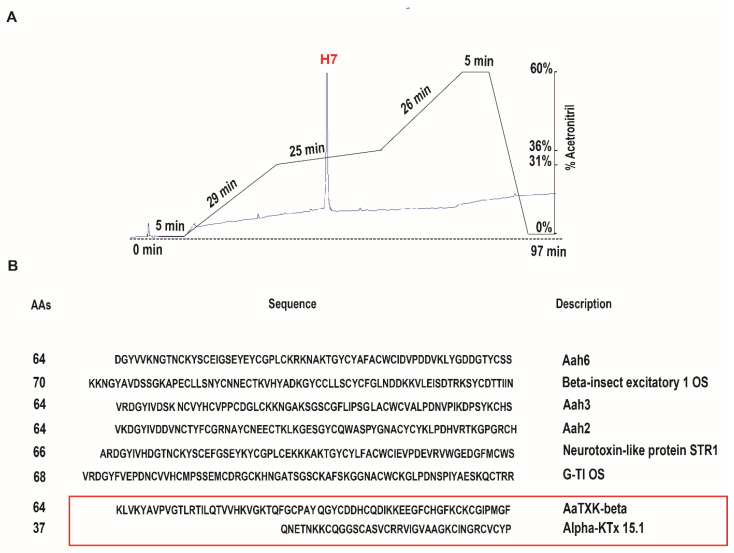
NanoLC/HRMS-MS analysis of H7. (**A**) Purification of H7 subfraction. The purification of fraction H7 was repeated on a C18 column with a modified gradient allowing to collect a unique sharp peak eluted at 38 min. (**B**) H7 fraction was subjected to disulfide bridges reduction and trypsin digestion, and the obtained peptide mixture was analysed by NanoLC/HRMS-MS. Data analysis led to the identification in the H7 fraction of Aah6, Beta-insect excitatory 1 OS, Aah2, Aah3, Neurotoxin-like protein STR 1, G-TIOS, alpha-KTx 15.1 and AaTXK-beta. Signal peptide and polypeptide sequences are not reported.

For a refined purification, fraction H7 was passed again through a C18 column with a modified gradient allowing to collect a unique sharp peak, which was further submitted to mass spectrometry (Figure 5A). H7 subfraction was subjected to disulfide bridges reduction and trypsin digestion (Promega, Madison, WI, USA), and the obtained peptide mixture was analysed by NanoLC/HRMS-MS. Putative amino acid sequences obtained were analysed against nucleotide sequences database dynamically translated in all reading frames (tBLASTn) from the Uniprot library of *Androctonus*. The correlation of the measured mass of the peptides and their sequences allowed to recognize eight known proteins (Figure 5B); including two beta-toxins (Aah6, Beta-insect excitatory 1 OS), 2 alpha-toxins (Aah2, Aah3), a non-toxic polypeptide (Neurotoxin-like protein STR 1), a Kunitz trypsin inhibitor (G-TI), an alpha-KTx (alpha-KTx 15.1) and a Beta-KTx (AaTXK-beta). Among these eight peptides, six of them (long toxins composed of 66 to 88 amino acids) are anti- Na^+^ channel toxins. Indeed, Aah6, Aah2, Aah3, AaH IT1, AaH STR 1, belong to the Na^+^ channel inhibitor family. They are characterized by a structural arrangement of an alpha-helix connected to antiparallel beta-sheets by four disulfide bonds, otherwise, only two of them are voltage gated K^+^ channel modulators, alpha-KTx 15.1 (37 amino acids) and AaTXK-beta (64 amino acids) (Figure 5B, Table 2). Since we have successfully identified the different peptides present in H7 and because native toxins are present in a low amount in crude venom, we decided to pursue the rest of the work, by using in silico study. Based on the structure-function relationship studies, on scorpion venom toxins [25,26], we suggest excluding anti-Na^+^ channels toxins and to predict how others might bind to Kv3.1 and whether they are responsible for IKv3.1 inhibition.

### 2.5. In Silico Study to Identify the Peptide Blocking Kv3.1

#### 2.5.1. Molecular Models of Alpha-KTx 15.1, AaTXK-Beta and Kv3.1 Channel

**Figure 6:**
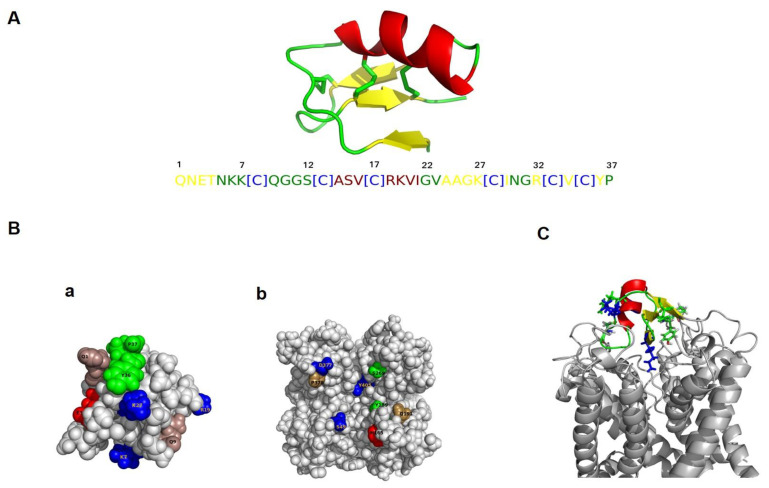
Molecular modeling of alpha-KTx 15.1 and Kv3.1 channel interactions. (**A**) 3-D structure model of alpha-KTx 15.1 adopts the common Csαβ fold that allows the molecule to have a stable three-dimensional conformation. alpha-KTx 15.1 sequence (37 amino acids), alpha-helix coloured in red, beta sheet in yellow, cysteine residues in green. (**B**) Hot spot amino acids (coloured) implicated in the interaction between alpha-KTx 15.1 (**a**) and Kv3.1 channel (**b**). (**C**) Docking complex of alpha-KTx 15.1/Kv3.1 showing that the beta sheet of the toxin interacts with the selectivity filter residues of the channel. The pore is physically blocked with the K27 lateral chain of alpha-KTx 15.1.

The Kv3.1 channel structure model was generated by homology to the crystal structure of the Kv1.2-Kv2.1 paddle chimera channel (PDB code 2R9R). The sequence of the human Kv3.1 K^+^ channel subtype was extracted from the Uniprot database under the accession number P48547 and contains 511 amino acids. We removed the segments S1, S2, S3 and S4 of Kv 3.1 channel prior to the docking. Indeed, AmmTX3 has the highest sequence identity (93%) with alpha-KTx 15.1. They differ by only two conserved amino acids (Q/Z) in position 1 and (N/I) in position 2 of their sequences (data not shown). Alpha-KTx 15.1 is a polypeptide chain of 37 amino acid residues. It has the characteristic folding of K^+^ scorpion toxins: a double stranded antiparallel beta sheet and an alpha helix, reticulated by three disulfide bonds. In fact, alpha-KTx 15.1 adopts the common Csαβ (Cysteine-stabilized α/β motif) fold (Figure 6A). AaTXK-beta belongs to the long chain scorpion toxin family and is a polypeptide chain of 64 amino acid residues cross-linked by only three disulfide bridges (Supplementary Material, Figure S1).

#### 2.5.2. Molecular Docking of Alpha-KTx 15.1 and AaTXK-Beta with Kv3.1 Channel

The main goal of the bioinformatic study is to identify which of the toxins, alpha-KTx 15.1 and AaTXK-beta toxin, blocks Kv3.1 channel. We used an in silico protein–protein docking to predict alpha-KTx 15.1 and AaTXK-beta with Kv3.1 channel mode of interaction. We found that AaTXK-beta does not interact with Kv3.1 channel (data not shown) contrary to the alpha-KxT15.1 that occludes the K^+^ channel pore (Figure 6C). Indeed, the top 10 docking solutions describe the same interaction mode. After visualization with pymol, we propose the best docking solution as a model of interaction between the alpha-KTx 15.1 toxin and Kv3.1 (Figure 6C) This complex presenting the best score shows an interaction mode in which the beta sheet of the toxin interacts with the selectivity filter residues. Lys27 of alpha-KTx 15.1 seems to play an important role in the interaction. The blocking of the pore is physically achieved by the means of the Lys27 lateral chain of alpha-KTx 15.1 interacting into the K^+^ channel selectivity filter (Figure 6C). Based on the molecular docking study, we predict several interactions of medium strength (2–6 Å). R19 and E3 are involved in the interaction and establish a salt bridge owing to Asp377 and His165 at 2.5 Å and 2.1 Å, respectively (Figure 6B). Several other amino acids of alpha-KTx 15.1 are involved in the interaction with the Kv3.1 K^+^ channel. Indeed Gln1/Gln191, Glu3/His165, Lys7/Ser49, Gln9/Pro378, Arg19/Asp377, Lys27/Tyr405, Tyr36/Tyr189 and Pro37/Ser269 of the complex toxin-channel (Figure 6B).

## 3. Discussion

This original study highlights the discovery of the first scorpion peptide inhibiting Kv3.1 channel. In fact, in the present work, we describe the biochemical and functional characterization of an *Androctonus australis hector* scorpion venom peptide that potently blocks Kv3.1 channel. To achieve our objective, we combined biochemistry, electrophysiology, high-resolution molecular spectrometry, and computational methods, based on different stages of findings: (1) AahG50 is a toxic venom fraction showing an inhibitory effect on Kv3.1 current amplitude with an IC50 of 50 µg/mL. (2) Among eighteen subfractions obtained by FPLC, only one (F5) is able to reproduce the AahG50 inhibitor effect whose IC50 is only about 5 µg/mL. (3) HPLC of F5 yields 18 peaks among which only the seventh (H7) inhibits the whole cell Kv3.1 current with IC50 equal to 1 µg/mL. (4) The blocking effect of H7 was confirmed on Kv3.1 single channel recordings. (5) H7 content analysis with NanoLC/HRMS-MS shows the presence of eight peptides and only two of them are voltage-gated K^+^ channels ligand, AaTXK-beta and alpha-KTx 15.1. (6) In silico studies demonstrate that only one toxin might block the Kv3.1. This toxin is alpha KTx15.1, a short 37 amino-acids peptide with one alpha helix, two stranded beta sheets and three disulfide bridges.

At the macroscopic level, H7 seems to act on the Kv3.1 channel as an open channel blocker such as fluoxetine [14], paroxetine [43] and psoralen [44] with the following properties: (1) the inhibition of Kv3.1 current is in the entire voltage range over which Kv3.1 channels are activated at potentials between −20 mV and +60 mV. (2) This induced inhibition is voltage dependent and increases steeply in the voltage range of channel activation (3) the toxin does not affect the threshold of channel activation (Thresh = −20 mV). Contrary to BDS-I and BDS-II, the first peptides able to inhibit Kv3 current [45] and gambierol, which instead bind to the resting state of the voltage sensor of Kv3.1 channels [46,47]. At the single channel level, H7 (0.6 µg/mL equivalent to 170 nM) strongly decreases the current amplitude and induces the channel closing supporting the data obtained in whole cell mode. By contrast to AUT1 (10 µM) that increases the single Kv3.1 channel activity at negative potentials [15].

The analysis of H7 with NanoLC/HRMS-MS accompanied by data mass mapping identified 8 known proteins. Six of them were previously classified and described, based on their structural features and their functional aspects, as Na^+^ channel toxins [25]. Only two were described as voltage-gated K^+^ channel modulators, an alpha-KTx (alpha-KTx 15.1) and a beta-KTx (AaTXK-beta). AaTXKβ was described as the first peptide activator of Kv7.4 channels which also acts as a subtype-selective activator of Kv7.3, Kv7.2/3, and Kv7.5/3 subunits [38]. Moreover, alpha-KTx 15.1 belongs to the alpha-KTx family. About half of the known 120 alpha-KTx have been tested directly against different K^+^ channels. The majority of the reported functions have been determined on A-type K^+^ channel [48,49,50], Shaker-related channels [39], Kv4 [51,52,53], hERG currents [42] or on the Ca^2+^-activated K^+^ channels [26]. The alpha-KTx 15.1 has a high affinity for transient K^+^ current [39,40,41,42,43,44,45,46,47,48]. The mechanism of blockade is in a simple bimolecular and pore-directed binding fashion, which resembles the mechanism that is described for other K^+^ channels by MacKinnon and Miller and Giangiacomo et al. using Charybdotoxin and Iberiotoxin, respectively [54,55].

However, the activity of AaTXKβ and alpha-KTx 15.1 on Kv3.1 has never been investigated. We used molecular modelling of the Kv3.1 channel to understand how they interfere with the Kv3.1 channel protein. The molecular docking shows that only alpha-KTx 15.1 occludes Kv3.1 channel pore contrary to AaTXK-beta that does not show any interaction with this channel.

Indeed, alpha-KTx 15.1 shows 94% sequence homology with AmmTX3 isolated from the venom of the scorpion *Androctonus mauretanicus* and 91% homology with BmTX3 from *Buthus martensi* which blocks at 0.1 μM A-type K^+^ currents in cerebellum granular cells and striatum cultured neurons, respectively [50,51,52]. We found that alpha-KTx 15.1 affinity to the whole cell Kv3.1 channel is in the same range, about 170 nM. The molecular docking study confirms that alpha-KTx 15.1 interacts with the S4 site in Kv3.1 channel selectivity filter owing to Lys27 located in its lateral chain, as previously suggested that the critical Lys27 protrudes into the pore of the channel [56] and the interaction is strengthened by the basic amino acid, Lys19, among alpha-KTx 15.1 sequence. In fact, alpha-KTx15 peptides define their targets more precisely, due to the “hot spot” composed of two basic residues Arg18 and Lys19, near the end of the α-helix [42]. Alpha-KTx 15.1 and Kv3.1 interaction is consolidated through KTX N-terminal pyroglutamic acid and Q191. It was previously shown that the generation of pyroglutamic acid at the N-terminus can greatly enhance the blocking effect of toxins for their targeted channels [57]. The computational study highlights also the importance of the C-terminal region of alpha-KTx 15.1 represented by Tyr36 and Pro37, that are involved in the interaction with the Kv3.1. It is, actually, known that members of alpha-KTx are dotted with a “hot spot” that interferes with K^+^ channel pore, qualified as the canonical dyad previously proposed to be necessary for blocking K^+^ channel conduction with high efficacy [41,42,43,44,45,46,47,48,49,50,51,52,53,54,55,56,57,58]. Moreover, a previous study showed that Tyr36 is a crucial amino acid required in the functional surface of BmTX3 to stabilize the toxin/receptor complex, via the aromatic ring [41].

This study provides new insight into the possible targets of alpha-KTx 15.1 on Kv3.1 channels, besides Kv4 channels type, suggesting its emerging potential therapeutic implications to be tested for the treatment of neuroinflammation and neurodegenerative disorders. Indeed, Kv3.1 channels have a remarkable role in the fast action potential repolarization abundant in rapidly firing neurons, such as the auditory brainstem, and hippocampal and cortical interneurons and play a critical role in the synchronization of cortical circuits and in the generation of rhythms. Kv3.1b is expressed in embryonic and perinatal neurons and their selective blockade increases NPC proliferation in vitro [9] that are to be used for the treatment of neurodegenerative disorders. Furthermore, suppressing Kv3.1 by 4-aminopyridine (4-AP) alters neural circuit activity, that may enhance brain derived neurotrophic factor (BNDF) signalling and hence protect axons from inflammatory insults [59]. Moreover, recent studies investigated the role of Kv3.1, as a new therapeutic target for cancer metastasis by inhibiting cell migration and invasion [60]. Further in vivo pharmacological tests should be performed to validate the therapeutical potential of alpha-KTx 15.1 when the toxin will be available in its pure form, including its toxicity and its pharmacokinetics dynamic.

In conclusion, we identified alpha-KTx 15.1 as the short toxin from Aah scorpion venom having the ability to inhibit, in a potent manner, the Kv3.1 channel by occluding the pore. It seems that this toxin inhibits 51 ± 0.2% of whole cell IKv3.1 at 1 µg/mL concentration. Its inhibitory effect is confirmed on Kv3.1 single channel recordings. In silico, studies reinforce this conclusion and elucidate the different sites involved by this active peptide in the complex formation and so, in the Kv3.1 blockade.

Further investigations include the generation of Kv3.1 mutants at the deduced interacting residues to validate the strong interaction of alpha-KTx 15.1 with Kv3.1 channel binding site. Otherwise, and since native toxins are present in a low amount in crude venom, including alpha-KTx 15.1, work is in progress to study the effect of synthetic alpha-KTx 15.1 analogs on Kv3.1 channel compared with the native toxin. In addition, studies may be performed on the specific affinity of these analogs on other Kv.3 channels family (Kv3.2 to Kv3.4) compared with Kv3.1 and with A-type K^+^ channels, especially Kv4.2 and Kv4.3 that are abundantly expressed in the brain [61,62]. These findings will give a deeper understanding of the specificity effect of alpha-KTX 15.1 as a peptidic tool useful for a structure-function relationship investigation of Kv3.1 and for developing a model of therapeutic biomolecules against diseases involving this channel.

## 4. Materials and Methods

### 4.1. Materials

Scorpion Venom: Venom of *Androctonus australis hector* was collected from Beni Khedache (Tunisia) by the veterinarian service of the Pasteur Institute of Tunis (Tunis, Tunisia) and was kept frozen at −20 °C in its crude form until use.

### 4.2. Methods

#### 4.2.1. Biochemistry

Venom Purification. Crude venom was dissolved in water and loaded on Sephadex G50 gel filtration chromatography columns (2_K26/100; Pharmacia; GE HealthCare, Velizy-Villacoublay, France). Columns were equilibrated and eluted with 0.1 M acetic acid buffer, pH 4.7. After freeze drying, the resolved fractions were stored at −20 °C until use. The elution profile of Aah was collected in five subfractions (M1, M2, AahG50, M3 and M4). The major fraction named AahG50, is the toxic one and contains toxins of 3000–7000 Da. After lyophilization, the AahG50 was fractionated by FPLC on a cation exchange Resource S pre-equilibrated with 0.05 M ammonium acetate pH 6.6. Proteins were eluted with a 60 min linear gradient from 0.05 to 0.5 M ammonium acetate, pH 6.6, at a flow rate of 0.8 mL/min. Absorbance was monitored at 280 nm. HPLC purification of the FPLC fraction was performed using a C18 reversed-phase HPLC column (5 mm, 4.6–250 mm, Beckman), equipped with a Beckman Series 125 pump and a Beckman diode array detector set. Elution was controlled by means of the GOLD software. Proteins were eluted from the column at a flow rate of 1 mL/min, using a multi-step gradient (90 min) from 0 to 60% of buffer B (0.1% TFA in CH3CN) in buffer A (0.1% TFA in water). Polypeptide concentration was determined using QuantiPro BCA Assay Kit (Sigma Aldrich, Darmstadt, Germany).

Protein Precipitation, Disulfide Bridges Reduction and Enzymatic Hydrolysis. Purified venom fraction was dissolved in 100 mM NH4HCO3 (Sigma Aldrich, Darmstadt, Germany), and dithiothreitol (Sigma Aldrich, Darmstadt, Germany) was added to a final concentration of 10 mM. Sample was allowed to react at 55 °C for 1 h. Iodoacétamide (375 mM) (Sigma Aldrich, Darmstadt, Germany) was added, and the sample incubated 30 min at 37 °C then acetone was added, and the sample was conserved at −20 °C overnight. Enzymatic digestion was then performed by adding trypsin (Promega, Madison, WI, USA), with an enzyme/substrate ratio of 1/50 *w/w* at 37 °C overnight. Formic acid was added at a final concentration of 5%, the sample was dried by speedvac and was conserved at −80 °C.

#### 4.2.2. Electrophysiology

- Two-Microelectrodes Voltage Clamp

Expression of Kv3.1 in *Xenopus laevis* oocytes: Experiments on *Xenopus* oocytes were carried out following the European Community Council Directive (2010/63/EU), for experimental animal care and procedures. The protocol of animal handling and oocytes extraction was approved by the Pasteur Institute of Tunis Biomedical Ethic Committee (Approval code 05/19; reference: 2018/39/I/LR16IPT08/V0). cDNA encoding for human Kv3.1b was cloned in the *Xenopus* oocyte expression vector pcDNA. After linearization with HpaI, capped cRNA was transcribed in vitro using the SP6 mMessage mMachine kit (Ambion, Foster City, CA, USA). *X. laevis* adult females were anesthetized with 0.17% solution of 3-aminobenzoic acid ethyl ester methane sulfonate salt (Sigma-Aldrich, Saint-Quentin Fallavier, France), and parts of ovaries were surgically removed from the abdominal cavity and bathed in sterile modified Barth’s solution (MBS) of the following composition (in mM): 88 NaCl, 1 KCl, 0.4 MgCl_2_, 2.4 NaHCO_3_, 0.8 MgSO_4_, 10 HEPES, 2.4 CaCl_2_, and 0.3 Ca(NO_3_)_2_, pH 7.4. *Xenopus* oocytes were then defolliculated enzymatically by incubation for 2 h in sterile MBS containing 2 mg/mL collagenase (type A and type B; Roche, Indianapolis, IN, USA), followed by three to four washes in MBS. Stage V and VI oocytes were injected with 50 nL of mRNA (10 ng/oocyte) using an automatic microinjector (Nanoject; Drummond Scientific, Broomall, PA, USA). Oocytes were kept at 18 °C in sterile MBS supplemented with 0.1 mM gentamicin until electrophysiological experiments were performed.

Measurements: two days after mRNA injection, the two-microelectrodes voltage-clamp measurements in *Xenopus* oocytes were performed at room temperature (22–24 °C) using a Geneclamp 500 B amplifier combined with Digidata 1440 A (Molecular Devices, Foster City, CA, USA). Micropipettes were pulled from borosilicate glass capillaries on a Flaming/Brown type pipette puller (P-97; Sutter Instruments, Novato, CA, USA) and had a tip resistance of 1–2 MΩ when filled with 3 M KCl. Data were filtered at 1 KHz, and voltage step protocols and current analysis were performed with pCLAMP 10 software (Molecular Devices). During the recordings, oocytes were perfused with MBS solution containing (in mM): 96 NaCl, 4 KCl, 1 MgC1_2_, 1.8 CaC1_2_, 5 HEPES, at pH 7.6, in a small chamber (1 mL volume). The perfusion system was controlled by a Manifold Solution Changer (MSC-200; Bio-Logic, Grenoble, France). Kv3.1 currents were induced by 250 ms depolarizing pulses from a holding potential of −80 mV, in the −70 to 70 mV range and 10 mV steps. Scorpion fraction activity was monitored using a single depolarizing step at +40 mV during 250 ms.

- Whole-cell patch-clamp

Cell transfection: Kv3.1 channels were expressed in Chinese hamster ovary (CHO) cells by transient transfection, using plasmid containing cDNA encoding human Kv3.1b cloned in pcDNA3 (Zhang, Yalan, Yale University School of Medicine, New Haven, CT, USA). According to the experimental protocol, these plasmids were expressed individually with a plasmid-expressing enhanced green fluorescent protein (GFP) used as a transfection marker. Total cDNA in the transfection mixture was kept at 1.5 µg. CHO cells were grown in 30 mm plastic Petri dishes in Dulbecco’s modified Eagle’s medium containing 10% fetal bovine serum, penicillin (50 U/mL), and streptomycin (50 mg/mL) in a humidified atmosphere at 37 °C with 5% CO_2_. The cells were transfected the next day with the appropriate cDNA using Lipofectamine 2000 (Life Technologies, Carlsbad, CA, USA), according to the manufacturer’s protocol. Electrophysiological experiments were performed 48 h after transfection.

Whole-cell recordings: currents from CHO cells were recorded at room temperature (24 °C) in whole-cell configuration with an EPC-10 amplifier (HEKA Electronic, Lambrecht, Germany). The media used within the pipette were, respectively (mM): 110 KCl, 5 NaCl, 2 MgCl_2_, 10 ethylene glycol-bis (2-aminoethyl ether)-N, N, N0, N0-tetraacetic acid (EGTA) and 5 mM HEPES and in the bath (mM): 100 N-methyl-D-Glucamine-Cl, 5 KCl, 2 MgCl_2_, 50 NaOH, 50 acetic acid and 5 HEPES at pH 7.3. Membrane currents were elicited from a holding potential of −80 mV, by depolarizations ranging from −120 to +60 mV. Scorpion fraction activity was monitored using a single depolarizing step at +40 mV for 250 ms. We used only cells with series resistance less than 5 MΩ for analysis. Patchmaster, Fitmaster (HEKA Electronic, Lambrecht, Germany) and IgorPro (WaveMetrics, Inc., Lake Oswego, OR, USA) software were used for data acquisition and analysis. Recording pipettes were from glass capillaries (Hematocrit, Modulohm A/S, DK). Their resistances were 1.5–4 MΩ and pulled with a PC-10 Narishige puller.

- Single channel recording

Principle: The Orbit mini system (Nanion Technologies, München, Germany) was used for single-channel recording. Kv3.1 channel proteins are expressed in planar lipid bilayers. The lipid bilayers were formed using 1,2-diphytanoyl-sn-glycero-3-phosphocholine (Avanti Polar Lipids, Alabaster, AL, USA) in the level of the four holes (100 µM) in 150 mM MOPS solution pH 7.4. Kv3.1 channels were expressed in CHO cells in presence of GFP by transient transfection. Fluorescence-activated cell sorting (FACS) was used to select GFP positive-cells. The purified Kv3.1 proteins were supplemented with n-Dodecyl β-D-maltoside (DDM) (Thermo Scientific, Strasbourg, France) detergent (1 µL) (0.015%) and added to a preformed bilayer.

Single-channel recording: Currents were recorded at −60 mV holding potential using Elements Data Reader (Nanion, München, Germany) and analysed using Clampfit (Axon Instrument Inc., Burlingame, CA, USA) software, sampled at 100 μs and filtered at 1.25 kHz.

#### 4.2.3. Data and Statistical Analysis

For Kv3.1 currents in *Xenopus* oocytes, whole-cell conductance (G) was calculated according to the following equation: G = I/(V − E_K_), where I is the steady state current measured at the end of each depolarizing step, V is the step potential, and E_K_ is the reversal potential for potassium, which was calculated to be −84.6 mV. Normalized conductance voltage plots were obtained by normalizing conductance (G) to maximal conductance (Gmax) and were fitted to a single Boltzmann distribution of the following form: G = Gmax/{1 + exp[(V − V_1/2_)/k]}, where V is the test potential, V_1/2_ is the half-activation potential, and k is the slope factor [63].

Statistical differences between data groups were performed using excel and were expressed as mean ± S.E.M. Differences were tested, using XLSTAT software, applying an unpaired two-tailed Student’s t test, assuming that the population follows a Gaussian distribution. Differences were considered statistically significantly different versus respective controls when *p* < 0.05.

#### 4.2.4. Proteomics

- Protein identification: NanoLC/HRMS-MS:

Ten microliters of the resulting supernatant were analysed using an ESI-Q Exactive Plus mass spectrometer coupled to an Ultimate 3000 RSLC Nano System (Thermo Scientific). Liquid chromatography was performed with an EASY-Spray Pepmap C18, 2 µm, 25 cm × 75 µm and 100 μm column. The flow rate was set at 0.3 μL/min with a 5–45% gradient of solvent B (80% acetonitrile, 20% water, 0.1% formic acid) against solvent A (0.1% formic acid, 100% water) for 240 min. For MS analyses, full-scan mass spectra were measured from 350 to 1500 *m*/*z* with an AGC (Automatic Gain Control) target of 3 × 10^6^ and a resolution of 70 K. A top 15 data-dependent method was used for MS/MS spectrum acquisition with an AGC target of 1 × 10^5^, a resolution of 35 K and a dynamic exclusion of 40 s.

All MS raw data files were analysed by Proteome Discoverer software 1.4 (Thermo Scientific) using the Sequest HT search engine against the Uniprot database (Version 2015_2). Precursor mass tolerance was set to 10 ppm and fragment ion tolerance was 0.02 Da. Carbamidomethylation of cysteine (+57.021 Da) was set as static modifications and oxidized Methionine as dynamic modification (+15.995 Da).

A decoy database search strategy was also used to estimate the false discovery rate (FDR) to ensure the reliability of the proteins identified and at least two peptides were required for matching a protein entry for its identification.

#### 4.2.5. Computational or In Silico Study

- Molecular modelling of alpha-KTx 15.1, AaTXK-beta and Kv3.1 potassium channel

The pairwise alignment of target sequences with the templates, identified from the Protein Data Bank (PDB) based on their sequence identity, was built with the Needlemane Wunsch algorithm implemented in EMBOSS [64]. We used the comparative modelling by satisfaction of spatial restraints implemented in the program MODELLER in its version 9.24 [65]. The Kv3.1 channel structure model was generated by homology to the crystal structure of the Kv1.2-Kv2.1 paddle chimera channel. The sequence of the human Kv3.1 K^+^ channel subtype was extracted from Uniprot database under the accession number P48547. The sequence of the human Kv3.1 K^+^ channel subtype contains 511 amino acids. The chosen template for model building of alpha-KTx 15.1 and AaTXK-beta is, respectively AmmTx3 from *Androctonus mauretanicus* (PDB code 6GGZ) [66] and Hge36 Scorpine-like Peptide from *Hadrurus gertschi* (PDB code 5IPO). Indeed, AmmTX3 has the highest sequence identity (93%) with alpha-KTx 15.1. They differ by only two conserved amino acids (Q/Z) in position 1 and (N/I) in position 2 of their sequences. Overall, 500 structures were generated for alpha-KTx 15.1 and Kv3.1 channel, respectively, by using the default parameters of the program. The generated conformations were assessed with the DOPE (Discrete Optimized Protein Energy), a based knowledge potential implemented in MODELLER [67], from which we selected the structure with the best score. Several conformational and energetic evaluation methods (Ramachandran Plot, ProsaII and verify 3D) were used to assess the quality of the models [68,69,70].

- Toxin-channel docking study

Docking approaches are firstly based on research into the various partner linkage methods, then on the selection of the most probable model according to certain specific criteria. In some cases, it is possible to orient the research by experimental, evolutionary, or statistical data (guided docking). In this research study, the strong homology between our toxin and AmmtX3 and by reference to AmmtX3 of interaction with Kv4.3 channel led us to a guided docking based on the functional dyad (Lys27-Tyr36) [71]. In order to predict a reasonable model of interaction between alpha-KTx 15.1 or ATXK-beta and Kv3.1, we used ClusPro software for protein–protein docking [72]. The docking surface is restricted to the extracellular surface of the Kv3.1 K^+^ channel. The server performs three computational steps as follows: (1) rigid body docking by sampling billions of conformations, (2) root-mean-square deviation (RMSD) based clustering of the 1000 lowest energy structures generated to find the largest clusters that will represent the most likely models of the complex, and (3) refinement of selected structures using energy minimization.

## Figures and Tables

**Table 1:** Analysis of AahG50, F5 and H7 on hKv3.1 channel activation kinetics.

	Control	AahG50	F5	H7
Midpoint potential (V_1/2_) (mV)	0.28 ± 0.06	11.32 ± 0.07	16.70 ± 0.26	12.22 ± 0.24
Slope factor (k) (mV)	10.91 ± 0.05	15.71 ± 0.07	13.44 ± 0.24	12.69 ± 0.19
Threshold potential (Thresh) (mV)	−20	−20	−20	−20

Mean midpoints of activation curves (V_1/2_) and the slope factor (k) before and after application of AahG50 (50 µg/mL), F5 (5 µg/mL) and H7 (1 µg/mL) on hKv3.1 expressed in CHO cells.

**Table 2:** Classification of the peptides contained in H7 according to their activities.

Toxins	Ion Channel Activity
Aah6	Anti-insect beta-toxins: bind in a voltage-independent manner at site-4 of Na^+^ channels and shift the voltage of activation toward more negative potentials [27].
Beta-insect excitatory 1 OS	Beta toxins: specifically active on the insect nervous system by affecting Na^+^ channel activation and promoting spontaneous and repetitive firing [28,29].
Aah2	Alpha toxins: bind in a voltage-independent manner at site-3 of Na^+^ channels (Nav) and block neuronal transmission. The toxin principally slows the inactivation process of TTX-sensitive Na^+^ channels [19,30,31,32,33]. AaH2 sterically occludes VSD4 activation by forming a number of interactions that serve to pin the S3-S4 loop and S4 helix into a deactivated conformation [23].
Aah3	Alpha toxins: binds in a voltage-independent manner at site-3 of Na^+^ channels (Nav) and inhibit the inactivation of the activated channels, thereby blocking neuronal transmission [34,35].
Neurotoxin-like protein STR 1 (50% similarity with Aah6)	Non-toxic polypeptide: active on Na^+^ channel [36].
G-TI	Kunitz trypsin inhibitor inhibits Na^+^ channel.
AaTXK-beta	Beta-KTx: peptide activator of Kv7.4, Kv7.3 and Kv7.2/Kv7.3 channels. [37,38].
Alpha-KTx 15.1	Alpha-KTx: inhibits transient K^+^ channels (IA-type current) by occluding the outer entry to the K^+^ conducting pore [39,40,41,42].

## Data Availability

Data available on request from the authors.

## Supplementary Materials

The following are available online at https://www.mdpi.com/article/10.3390/ijms222212290/s1.
