# Supplementary material for: Cross Pharmacological, Biochemical and Computational Studies of a Human Kv3.1b Inhibitor from Androctonus australis Venom
Source: Int J Mol Sci. 2021 Nov 13;22(22):12290. doi: 10.3390/ijms222212290 (PMC8618407; doi:10.3390/ijms222212290)
Supplement: Supplementary file 1 [file ijms-22-12290-s001.zip › ijms-1425888-supplementary.pdf]

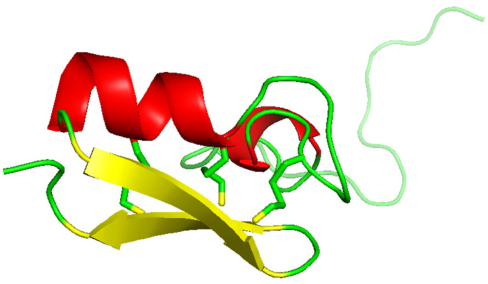

Figure S1: 3-D structure model of AaTXK-beta adopts the common Csαß fold that allows the molecule to have a stable three-dimensional conformation. Alfa-helice is colored in red, beta sheet in yellow, loop and cysteine residues in green.
